# Rule Following Mitigates Collaborative Cheating and Facilitates the Spreading of Honesty Within Groups

**DOI:** 10.1177/0146167220927195

**Published:** 2020-06-17

**Authors:** Jörg Gross, Carsten K. W. De Dreu

**Affiliations:** 1 Leiden University, The Netherlands; 2 University of Amsterdam, The Netherlands

**Keywords:** honesty, norms, rule-following, social conformity, collaborative cheating

## Abstract

Compared with working alone, interacting in groups can increase dishonesty and give rise to collaborative cheating—the joint violation of honesty. At the same time, collaborative cheating emerges some but not all of the time, even when dishonesty is not sanctioned and economically rational. Here, we address this conundrum. We show that people differ in their extent to follow arbitrary and costly rules and observe that “rule-followers” behave more honestly than “rule-violators.” Because rule-followers also resist the temptation to engage in collaborative cheating, dyads and groups with at least one high rule-follower have fewer instances of coordinated violations of honesty. Whereas social interaction can lead to a “social slippery slope” of increased cheating, rule-abiding individuals mitigate the emergence and spreading of collaborative cheating, leading to a transmission advantage of honesty. Accordingly, interindividual differences in rule following provide a basis through which honest behavior can persist.

## Introduction

People like to see themselves as moral beings (Abeler et al., 2014; Jordan et al., 2011; Mazar et al., 2008), and care about what others think of them (Utikal & Fischbacher, 2013). As a result, individuals often abide by societal rules, try to live up to ethical standards, and adapt their behavior to fit culture-specific norms and rules of engagement (Gelfand et al., 2011). Yet, people also frequently engage in selfish or unethical behavior and deviate from social norms and moral principles (Fischbacher & Föllmi-Heusi, 2013; Heck et al., 2018; Shalvi et al., 2015). In short, individuals are often torn between pursuing selfish goals on the one hand, and abiding by rules and norms that restrict this pursuit of personal self-interest on the other hand.

Whereas one may expect individuals to engage in dishonesty especially when they are operating in social isolation, research in psychology and economics rejects this intuition. Unethical behavior such as cheating appears particularly prominent when people operate in groups rather than alone (Cohen et al., 2009; Conrads et al., 2013; Falk & Fischbacher, 2002; Gino et al., 2013; Kocher et al., 2017). For example, dishonesty increased after witnessing one group member ostentatiously cheat for personal gain, leading to a “social” slippery slope of increasing dishonesty (Gino et al., 2009; see also Diekmann et al., 2015; Köbis et al., 2017; Soraperra et al., 2017). Likewise, Weisel and Shalvi (2015) observed that individuals systematically coordinate cheating behavior. In a dyadic die-rolling task, two individuals privately threw a die and earned money if they reported to have thrown the same number. Participants reported to have thrown a “double” 82% of the time, a number that not only exceeds chance (16.6%) but also what individuals reported when having the same monetary incentive to lie but acting alone. This form of collaborative cheating (that the authors also coin “corrupt collaboration”) is reminiscent of the widely publicized scandals in the automotive and financial industries in which professionals initiate and reinforce each other’s dishonest behavior within their group or organization (e.g., Enron or Diesel scandal; Goodman, 2015; also see Gino et al., 2013; Wiltermuth, 2011).

The emergence and persistence of collaborative cheating raises the question how honesty can prevail in groups or society at large. How can we reconcile that group interaction can provide fertile grounds for collaborative cheating while honesty and fairness is valued? What mechanism explains that honesty is adhered to and persists across societies even when norm violations and acts of dishonesty are serving personal interests, are difficult to monitor, and are formally not sanctioned? Here, we address this conundrum by taking a person × situation perspective. We build on previous findings that showed that individuals differ in their propensity to follow rules, even when rules are arbitrary and abiding by them is financially costly. We show that “rule-followers” are more resilient to social influence than “rule-violators.” Because collaborative cheating takes “two to tango,” one individual scoring high on rule following mitigates the emergence of coordinated lying.

### Individuals Differ in Their Propensity to Follow Rules

Distinct lines of research converge on the idea that humans often comply with rules and regulations. Whether we consider work on obedience to authority (Milgram, 1963), social conformity and norm compliance (Stubbersfield et al., 2019), or adherence to culture-specific rules such as “wait until the light turns green,” or “thou shall not cheat” (Gelfand et al., 2011), people often abide by rules. It has been argued that deviations from rules generate a psychological cost (Abeler et al., 2014; Gross & De Dreu, 2017; Kimbrough & Vostroknutov, 2016, 2018; Krupka & Weber, 2013) and create a cognitive conflict between what one should and wants to do (Pfister et al., 2019; Schelling, 1984).

Yet, people also vary in the extent to which they obey authority (Gross et al., 2018a; Haslam & Reicher, 2012), conform to group norms (Hodges & Geyer, 2016), or stick to rules and regulations (Bicchieri, 2005; Bicchieri et al., 2020). Similarly, people differ in cheating rates and their willingness to lie for profit (Heck et al., 2018; Hilbig & Zettler, 2015). These disparate findings combined suggest that people systematically differ in the value they derive from abiding by rules and/or the disutility incurred when violating rules (Gross & De Dreu, 2017; Kashy & DePaulo, 1996; Kimbrough & Vostroknutov, 2016, 2018). Indeed, others before us have shown that individual differences that are conceptually or empirically related to rule following, such as guilt proneness and honesty–humility, correlate with ethical conduct and shying away from delinquent behavior (Fleeson et al., 2014; Heck et al., 2018; Hilbig & Zettler, 2015).

Whether and how interindividual differences in rule following may shape social interactions, and in particular the extent to which group members engage in collaborative cheating, is open to debate. One possibility is that groups engage in collaborative cheating already when at least a few individuals have low rule-following propensity (the proverbial rotten apple that spoils the barrel), and are drawing their more honest rule-following partners into increasingly dishonest behavior. This would resonate with the observation of increased cheating rates in teams and groups compared with individuals (Cohen et al., 2009; Conrads et al., 2013; Falk & Fischbacher, 2002; Gino et al., 2009, 2013; Kocher et al., 2017; Weisel & Shalvi, 2015). It would also imply that rule-followers adapt to the dishonesty of rule-violators rather than the other way around, leading to a crowing out effect of honesty, similar to how selfishness of few individuals can crowd out cooperation in social dilemmas (e.g., Gross et al., 2016).

An alternative possibility, however, is that collaborative cheating may fail to emerge and honesty persists already when few group members have a high propensity to follow rules. First, rule-followers may be more resilient to social influence and do not go along with other’s deceitful initiatives and, second, displays of honesty by rule-followers may signal that cheating is not acceptable. Evidence pointing to this latter possibility comes from research showing that people not only systematically differ in the extent to which they cheat (Cohen et al., 2012; Fleeson et al., 2014; Heck et al., 2018; Hilbig & Zettler, 2015) but also that some people are better at resisting selfish temptations (Gino et al., 2011) and are more resilient to social influence (Trevino, 1986). Furthermore, appealing to ethical norms can increase honesty (Cohn et al., 2014; Hallsworth et al., 2017), and cheating seems highly malleable to monitoring and the possibility that lies are detected (Rosenbaum et al., 2014). Possibly, being confronted with a rule-follower may also increase self-awareness, which has been shown to decrease cheating (Diener & Wallbom, 1976). Because collaborative cheating requires to coordinate unethical misconduct, already one rule-follower may be able to successfully inhibit its success. Evidence for this latter possibility would point to rule following as a mechanism underlying the “stickiness” of honesty, explaining why honesty can persist even when local group interactions offer opportunities for self-serving and coordinated dishonesty. We return to this upon reporting the results of Experiment 1.

## Experiment 1

In Experiment 1, we empirically tested whether interindividual differences in a behavioral rule-following task (Gross & De Dreu, 2017; Kimbrough & Vostroknutov, 2016, 2018) predict honesty when cheating is tempting and not formally punished. In this task, participants have to place balls in one of two buckets. One bucket generates more income than the other bucket, yet the rule given to participants is to place the balls in the less profitable bucket. Even when the rule is strictly arbitrary, not justified, and no monitoring or formal sanctioning is present, some participants go along with the rule and earn less money from the task, whereas others persistently violate the rule and benefit financially (Gross et al., 2018a; Kimbrough & Vostroknutov, 2016, 2018; Thomsson & Vostroknutov, 2017). Behavioral rule following in this simple task correlates with personal need for structure (Gross & De Dreu, 2017), predicts normative behavior in social dilemma situations, and respecting norms such as trust and pro-sociality (Kimbrough & Vostroknutov, 2016, 2018). A recent brain stimulation study also revealed that rule following is causally linked to the right lateral prefrontal cortex, a brain region that has been associated with value-based cost–benefit decisions (Gross et al., 2018a).

Honesty was measured using the die-roll task, in which each participant throws a die under a cup—so that only he or she can see the number thrown—and reports the outcome for which the participant gets paid depending on the reported number (Fischbacher & Föllmi-Heusi, 2013; Shalvi et al., 2011). Because the die-roll is strictly private, participants can dishonestly report a higher number than they actually threw. Lying can be detected from significant deviations from the probability of each number being thrown (i.e., 16.7%; Fischbacher & Föllmi-Heusi, 2013; Gerlach et al., 2019). Earlier work revealed that lying in the die-roll task is correlated with unethical behaviors such as not paying for public transport (Dai et al., 2017), being absent from work without a reason (Hanna & Wang, 2017), not returning undeserved pay (Potters & Stoop, 2016), misbehaving in school (Cohn & Maréchal, 2017), and diluting milk with water in a dairy market (Kröll & Rustagi, 2017).

Both the rule-following task and the cheating task confront participants with a situation in which a rule prohibits to obtain monetary reward. Based on this conceptual similarity and results from previous research, it follows straightforwardly that rule following should predict honesty. Still, we deemed it important to establish such a link empirically for two reasons. First, previous research has shown that behavioral rule-following is correlated with normative behavior in social dilemma situations and prosocial choice, but has not investigated honesty in a cheating task. Second, rule-following is explicitly based on a demand effect—people are told to follow a specific rule—whereas rule violations such as lying in the die-rolling task are more obfuscated and people are not directly primed with the possibility that they could lie for profit.

### Method and Materials

#### Participants and ethics

We invited 70 subjects for a two-part experiment. Sample size was determined assuming a medium effect size (*d* = 0.3), based on previous data on interindividual difference measures and strategic behavior in economic games (Gross & De Dreu, 2019; Gross et al., 2018b; Hilbig & Zettler, 2015) with β = .8 and α = .05, resulting in a target sample size of *N* = 64 (based on G-Power 3.1; Faul et al., 2007).

The study received ethics approval from our Psychology Ethics Board. Subjects provided written informed consent and were debriefed upon completion of the studies. The experiments did not involve any deception and subjects were paid for participating at 6.50 €/hr and for their decisions during the experimental tasks. After the first task, participants were scheduled for a second appointment based on the experimental schedule and availability of the subject. Five participants failed to show up for their second appointment and did not respond to our contact attempts (dropout rate of 7%). Importantly, first-part behavior (measured rule following) of responders and nonresponders did not differ, two-sample *t* test, *t*(68) = 0.29, *p* = .78, two sided. Results are based on all participants who took part in both parts of the studies (*n* = 65, 57 female, *M*_age_ = 20.80 years, *SD*_age_ = 3.51 years).

#### Experimental procedures

Upon arrival, participants were assigned to an individual cubicle with a computer in front of them displaying the instructions. In the first part of the experiment, subjects engaged in the incentivized rule-following task (Kimbrough & Vostroknutov, 2016, 2018). Each subject had to place 30 balls in either a blue or yellow bucket on a computer screen. It was explained that for each ball they put in the blue bucket, they would receive €0.05 and for each ball they put in the yellow bucket they would receive €0.10. They then read that “the rule is to put the balls in the blue bucket.” Hence, following the rule was costly, whereas violating the rule was beneficial for the subject, creating a conflict between following rules and maximizing monetary payoff. As in previous studies, no reason was given for following the rule, and participants would not face any negative consequences for violating the rule (Gross & De Dreu, 2017; Kimbrough & Vostroknutov, 2016, 2018; Thomsson & Vostroknutov, 2017). We thus measured tendencies to follow rules in the absence of external enforcement (i.e., punishment or reward).

Participants were reinvited after 8.4 days on average (see supplemental material for further details). Prior to participation, participants only knew that the second part of the experiment would take 30 min. In the second part, each participant was again assigned to an individual cubicle with a computer and was given a die and a cup. Their task was to throw the die for six consecutive rounds, each time looking at the outcome and reporting the outcome to the computer (Fischbacher & Föllmi-Heusi, 2013). It was explained to participants that one random round would be selected by the computer at the end of the task and the participant would earn half of the reported outcome worth in euro (i.e., if the participant reported a 1, they earned €0.50 extra; for a 2, they earned €1 extra, and so on). Hence, there was an incentive to misreport the die-rolls to maximize additional earnings. Participants were informed that they would be paid the sum of money they earned across both sessions only after the second session, that experimenters could not monitor their behavior in the first or second session, and only had access to the total sum of money participants earned to pay participants.

### Results

**Figure 1.**
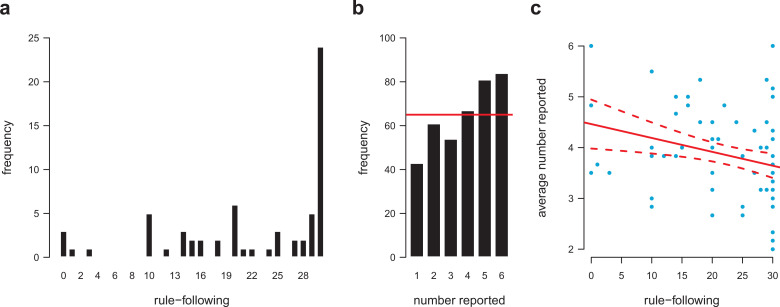
Rule following and honesty. Extent of rule following based on 30 balls for which participants had to decide whether to place each of them according to a costly rule or not (a) and distribution of die-roll reports in the second part of Experiment 1 (b). Red line indicates the distribution expected by chance (i.e., honesty). The extent of rule following in Part 1 and average die-roll reports, as a proxy for cheating, was negatively correlated (c). Red line shows the linear regression fit. Dotted lines indicate the 90% confidence interval of the regression fit.

Many participants actually followed the rule of the rule-following task completely, despite the economic incentive to do the opposite (Figure 1a). There also was considerable variability in rule following across participants (*M* = 22.20, *SD* = 9.21). Figure 1b shows that the distribution of die-roll reports deviated from what would be expected if people reported their outcome honestly, *M* = 3.86, one-sample *t* test, *t*(64) = 3.15, *p* = .003, two sided.

As predicted, rule following was significantly correlated with the reports in the die-rolling task obtained about 1 week later (Spearman *r* = −.29, *p* = .02, two sided). The more people followed the rule, the lower their average reported die-roll outcome (Figure 1c): Rule following predicted the degree of dishonesty.

## Discussion and Introduction to Experiment 2

Having established that individuals differ in rule following, and that rule following predicts future honesty, we asked how “sticky” rule following-based honesty is in social interactions. Experiment 2 combined the rule-following task with the incentivized dyadic die-rolling task (Weisel & Shalvi, 2015) to investigate whether people with a lower propensity to follow rules adapt to the behavior of people with a higher propensity to follow rules, or vice versa. In the dyadic die-rolling task, coordinated cheating by misreporting individual die-rolls is financially beneficial for both parties, whereas honesty is costly.

As noted at the outset, one possibility is that rule following predicts general adherence to societal norms, including those asking people to behave honestly, but not when in close interaction with others. Whereas rule-followers may not initiate cheating, they may still be sensitive to social influence and local expectations for self-serving behavior. Thus, if social interactions draw even rule-abiding individuals into violating rules of honesty, we should see low levels of collaborative cheating only when groups are composed of rule-following individuals, and to emerge already when one group member has a low propensity to follow rules (and invites collaborative cheating). Alternatively, it may be that more rule-abiding individuals are not only more honest but also resist social influence, preventing collaborative cheating to emerge and develop. Indeed, societies often construe abiding by norms as a reflection of moral development or character (Bicchieri, 2005), which may help individuals to shield themselves from peer influence and temptations (Fleeson et al., 2014; Ścigała et al., 2018). If true, low rule-abiding individuals should show the opposite pattern. While having a higher willingness to cheat for profit in social isolation, as we saw in Experiment 1, rule-violators should be more susceptible to social influence and adapt to displays of honesty. This we tested in Experiment 2.

### Method and Materials

#### Participants and ethics

We invited 136 subjects for a two-part experiment. The study received ethics approval from our Psychology Ethics Board. Because a priori power calculations for multilevel repeated-measure designs are complex and depend on many a priori assumptions (Gelman & Hill, 2006), sample size was determined based on the number of independent observations per condition (group level) in previous literature with similar design features (Abbink, 2004; Gino et al., 2009; Gross et al., 2018b; Weisel & Shalvi, 2015).

Subjects provided written informed consent and were debriefed upon completion of the study. The experiment did not involve any deception and subjects were paid for participating at 6.50 €/hr and for their decisions during the experimental tasks. After the first task, participants were scheduled for a second appointment based on the experimental schedule and participant availability. Eighteen participants failed to show up for their second appointment and did not respond to our contact attempts (dropout rate of 13%). Importantly, first-part behavior of responders and nonresponders did not significantly differ, two-sample *t* test, *t*(134) = 0.56, *p* = .58, two sided. Results are based on all participants who took part in both parts of the studies (*n* = 118, 93 female, *M*_age_ = 22.42 years, *SD*_age_ = 4.50 years).

#### Experimental procedures

**Figure 2.**
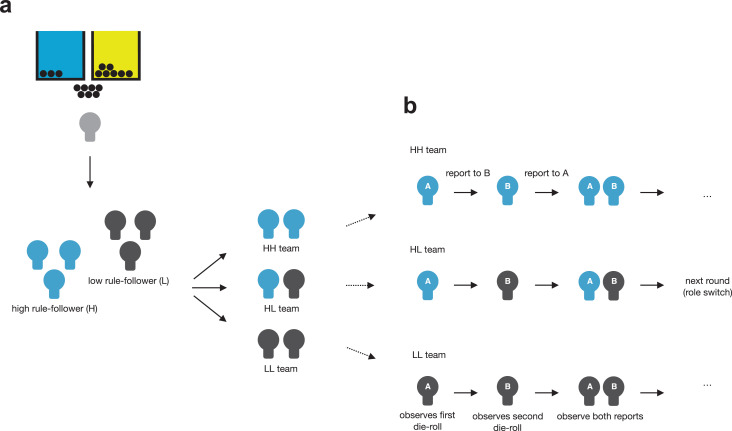
Experimental design. In the first part (a), participants were confronted with a costly rule that demanded to put each ball into the blue rather than the yellow bucket. Participants were categorized as high (H) or low (L) rule-followers based on the number of balls participants allocated according to the rule. In Experiment 2, they were then assigned to pairs of high rule-followers (HH teams), low rule-followers (LL teams), or mixed pairs of one high and one low rule-follower (HL teams). Participants were reinvited in these teams of two and played the dyadic die-rolling task (b). In this task, the first mover (A) rolls a die and reports the outcome to the second mover (B). The second mover then rolls an independent die and reports the outcome to A. If they both report to have thrown the same number (a “double”), they get paid according to the value of the double. After each round, the role (first mover/second mover) is switched.

As in Experiment 1, participants first performed the rule-following task (see procedure of Experiment 1). Participants were reinvited in pairs after around 2 weeks, on average. Pairs were constructed as follows. Based on the median rule following of the sample (median = 20 balls), we split the sample into “high” (H) and “low” (L) rule-followers. Participants were reinvited either in pairs of two high rule-followers (HH teams, *n* = 42), two low rule-followers (LL teams, *n* = 38), or mixed pairs comprised of one person scoring high and one person scoring low on rule following (HL teams, *n* = 38; Figure 2a). Prior to participation, participants only knew that the second part of the experiment would take 60 min and that they would interact with another participant. Each team engaged in the dyadic die-rolling task (Figure 2b). In this task, each team member privately rolls a die and is told to report the outcome of their die-roll to the computer. If the team reports to have rolled exactly the same number (a “double”), they get paid according to the worth of the double. For example, if both team members reported to have rolled a “1,” they got paid 1€ split equally among them. If they both reported a “6,” they got paid 6€ split equally among them. If they did not report a double (e.g., one reports a “1” and the other reports a “6”), they did not receive any payment. Hence, there was an incentive to coordinately misreport the die-rolls to maximize profits.

To further examine whether (a) a team of high rule-followers (HH) lies less than a team of low rule-followers (LL), and (b) whether in teams with mixed types, high rule-followers influence the behavior of low rule-followers more than the other way around, the dyadic die-rolling task was played sequentially (Figure 2b). Specifically, in each round, there was a first mover and a second mover. The first mover reported her die-roll first. The second mover then saw the report of the first mover on her computer screen before rolling her die. Then, the second mover reported her die-roll to the computer. The first mover, not knowing what the second mover would report, can “set the stage” and determine the potential worth of a double by lying (e.g., reporting a six for maximum potential gain). The second mover, knowing the report of the first mover, can then “get the job done” by matching the reported number of the first mover (e.g., also reporting a six). As such, the first mover cannot guarantee any payoff by lying, whereas the second mover cannot determine the value of a double but can guarantee a payoff above zero by matching the reported number of the first mover. At the end of each round, both participants received a summary showing the reported die-rolls and the payoff for this round (see supplemental material for details on the computer interface and instructions). After each round, the role of first and second movers was switched and each team interacted for 12 rounds. The first mover in Round 1 was determined randomly. Similar to Experiment 1, participants were paid based on their decisions in the rule-following task and one randomly selected round from the dyadic die-rolling task, in addition to their participation fee. Participants were paid the sum of money they earned across both experimental sessions after the second session. Experimenters could not monitor the behavior of participants in the first or second session and only had information about the sum of money that a participant earned after the second session to be able to pay participants.

#### Analyses

Because individual data points are clustered in individuals (across time) and teams (influence of partner), we fitted Bayesian multilevel regression models using STAN (Carpenter et al., 2017) as implemented in the brms R-package (Bürkner, 2017) for our two dependent variables: reported die-roll reports and doubles. Consequently, we report the 95% Bayesian confidence interval (CI) instead of *p* values for these models. Note that, because we used noninformative priors (see below), a 95% Bayesian CI that only contains negative or positive values can be interpreted as significant at a *p* = .05 two-sided threshold from a frequentist perspective. Using a frequentist approach based on maximum likelihood estimation (as implemented in the lme4 package in R) did not change our conclusions, but we decided to fit Bayesian models because they are widely recommended for multilevel modeling (Browne & Draper, 2006; Carpenter et al., 2017) and allowed us to restrict the variance of the dependent variable via priors. Die-roll reports were modeled as hierarchically clustered in subjects (Level 2) and teams (Level 3), estimating two hierarchically clustered random intercepts (see Equation 1).

$$y_{ijk}={\text{β}}_{0jk}+{\text{β}}_{1}X_{1ijk}+e_{ijk},e_{ijk}∼N\left(0,\sigma_{2}\right)\left(\text{Level 1}\right)$$

1 $${\text{β}}_{0jk}={\text{β}}_{0k}+e_{0jk},e_{0jk}∼N\left(0,\sigma_{e_{0jk}}^{2}\right)\left(\text{Level 2}\right)$$

$${\text{β}}_{0k}={\text{β}}_{0}+e_{0k},e_{0k}∼N\left(0,\sigma_{e_{0k}}^{2}\right)\left(\text{Level 3}\right)$$

where k = team, j = subject, i = decision.

Because changes in die-roll reports are restricted to lie between −6 and 6, we used noninformative uniform priors for each predictor (min = −6, max = 6) forcing the parameters in the model to respect the fact that the true average responses have to lie within this range. For double reports, we fitted a Bayesian multilevel logistic regression because the dependent variable is binary. Furthermore, for double reports, we only have one observation per team and round and hence only needed to estimate one random intercept for each team.

For each model, we used four chains with 10.000 iterations (5.000 burn-in iterations). For group-level effects of each grouping factor, we used the default option of the prior function, which restricts the variance to be nonnegative and following a half student *t* distribution with three degrees of freedom and a scale parameter that depends on the standard deviation of the response variable. The Gelman–Rubin statistic was below 1.05 for all parameters, indicating good mixing of the chains and thus high convergence.

### Results

As in Experiment 1, subjects rolled their die in private, prohibiting us to determine whether individual reports were honest or not. However, average die-roll reports were significantly above what would be expected by chance, *M* = 3.88, one-sample *t* test, *t*(58) = 4.89, *p* < .001, two sided, indicating that subjects engaged in lying to some degree.

#### Reported numbers

**Figure 3.**
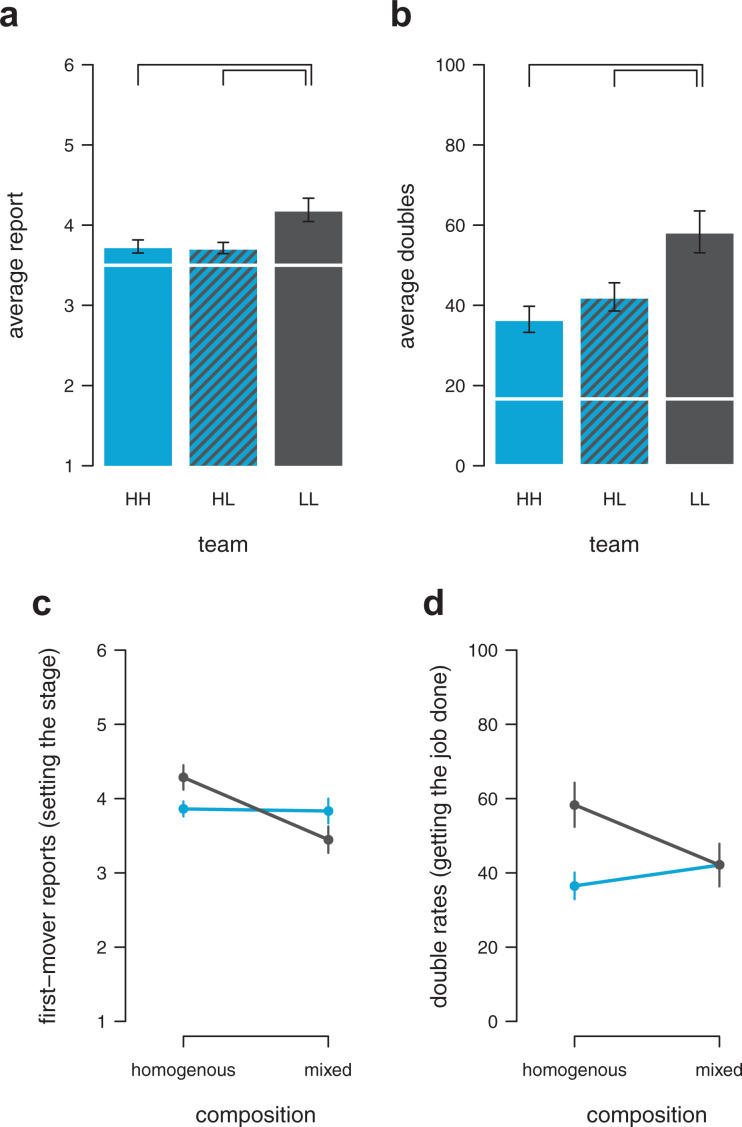
Rule following and collaborative cheating in pairs. Average die-roll reports (a) and average percentage of doubles (b) across conditions. HH = pair of two high rule-followers, LL = pair of two low rule-followers, HL = team of both types. White line indicates expectation based on chance (i.e., full honesty). Black connection lines indicate mean differences for which the Bayesian 95% confidence intervals do not overlap. Average die-roll reports of first movers (“setting the stage”; c) and percentage of matching the number reported by the first mover (“getting the job done”; d) of low rule-followers (black) and high rule-followers (blue) when interacting with the same type (homogeneous pair) or with the opposite type (mixed pair). Error bars show the standard error of the mean.

A team of low rule-followers (LL) systematically reported higher numbers (*M* = 4.19) compared with a team of high rule-followers (HH; *M* = 3.73, Figure 3a, Bayesian multilevel regression, HH vs. LL, *b* = 0.46, 95% CI = [0.11, 0.81]; Table 1). The reported die-roll outcomes of mixed teams (HL; one high type interacting with one low type, *M* = 3.71) were systematically lower compared with LL teams (Bayesian multilevel regression, HL vs. LL, *b* = −0.48, 95% CI = [−0.84, −0.12]). On the contrary, mixed teams were statistically indistinguishable from high rule-follower teams (Figure 3a, Bayesian multilevel regression, HH vs. HL, *b* = −0.02, 95% CI = [−0.38, 0.34]; Table 1). This pattern speaks to the possibility that high rule-followers influenced low rule-followers rather than the other way around. Importantly, however, changes in average reported numbers across team compositions cannot unequivocally be interpreted as a sign of social influence and should be interpreted with caution as the following thought experiment demonstrates. Assuming that LL teams would perfectly coordinate on reporting the highest number possible, we would expect an average die-report of LL = 6. Assuming that HH teams would stay perfectly honest, we would expect an average die-report of HH = 3.5. If we further assume that high and low types do not change their behavior in HL teams, we would expect an average report of (6 + 3.5) / 2 = 4.75 when the low type is in the first-mover position, reporting a 6. When the low type is in the second-mover position, we would expect an average report of (3.5 + 3.5) / 2 = 3.5 (the high rule-follower, as a first mover, reports honestly, and the low rule-follower, as the second mover, always matches the first mover’s number). Across rounds, we would hence expect an average report of HL = (3.5 + 4.75) / 2 = 4.125 in mixed teams. Note that this number is closer to the HH expectation than the LL expectation: e(HL) − e(HH) = 4.125 − 3.5 = 0.625 < 1.875 = 6 − 4.125 = e(LL) − e(HL). In other words, even without any social influence taking place between types, we would expect lower average die-reports in mixed teams. The measure of average die-reports can, hence, only provide some preliminary evidence that high rule-followers do not start to imitate low rule-followers, but not the other way around. To show that high rule-followers actually influenced the behavior of low rule-followers, we need to investigate double rates (for which the expectation of HL teams based on the above outlined assumptions lie in the middle of HH and LL teams, see supplemental material for a derivation) and look at the individual-level behavior of types across the first-mover and second-mover position.

**Table 1.** Random-Effects Regression Predicting Die-Roll Reports Based on Team Composition.

			95% CI	
Coefficient	Estimate	*SE*	L	U
Intercept (HH teams)	3.49	0.15	3.20	3.78
HL teams	−0.02	0.18	−0.38	0.34
LL teams	0.46	0.18	0.11	0.81
Round	0.04	0.01	0.01	0.06
σ_level 1_	1.62	0.03	1.56	1.68
σ_level 2_	0.14	0.09	0.01	0.34
σ_level 3_	0.44	0.07	0.30	0.59

*Note.* HH = pair of two high rule-followers; HL = mixed pair of one person scoring high on rule following paired with one person scoring low on rule following; LL = pair of two low rule-followers. σ refers to the error term on the individual-decision level (Level 1), subject level (Level 2), or team level (Level 3) following Equation 1.

#### Doubles

Teams containing two people scoring high on rule following (HH) reported to have thrown a double in 37% of the rounds. Teams containing two low rule-followers (LL) reported doubles in 58% of the rounds on average, an increase of 21% (Figure 3b, Bayesian multilevel regression, HH vs. LL, *b* = 1.11, 95% CI = [0.44, 1.81]; Table 2). The reported double rate of mixed teams (42%) was lower than the double rate of low rule-follower teams (58%; Bayesian multilevel regression, HL vs. LL, *b* = −0.84, 95% CI = [−1.55, −0.16]). However, the likelihood of reporting a double in mixed teams was statistically indistinguishable from high rule-follower teams (42% vs. 37%; Figure 3b, Bayesian multilevel regression, HH vs. HL, *b* = 0.27, 95% CI = [−0.40, 0.94]; Table 2). The results provide evidence that high rule-followers were able to increase honesty in low rule-followers and mitigate the extent of collaborative cheating. This can be achieved through two paths. First, reporting high numbers and “setting the stage” as a first mover may not pay off anymore, once paired with a high rule-follower who is unwilling to “play along.” This is a direct path of how high rule-followers may discourage lying of low rule-followers. In the second-mover position, low rule-followers can guarantee payoff by simply matching the reported number of the first mover. Yet, high rule-followers can influence the potential payoff by “setting a lower stage” (i.e., through honest reporting). We call this an indirect path of how high rule-followers may discourage lying of low rule-followers, because a low rule-follower should still match any number reported by a high rule-follower in the second-mover position from a rational money-maximization perspective.

**Table 2.** Random-Effects Logistic Regression Predicting Double Reports Based on Team Composition.

			95% CI	
Coefficient	Estimate	*SE*	L	U
Intercept (HH teams)	−0.92	0.29	−1.50	−0.36
HL teams	0.27	0.34	−0.40	0.94
LL teams	1.11	0.35	0.44	1.81
Round	0.04	0.03	−0.01	0.09
σ	1.18	0.17	0.88	1.52

*Note.* HH = pair of two high rule-followers; HL = mixed pair of one person scoring high on rule following paired with one person scoring low on rule following; LL = pair of two low rule-followers. σ refers to the error term on the team level. There is no individual-decision and subject-level error term because of the logistic regression model and because reported doubles are measured on the dyadic level.

We found evidence that high rule-followers influenced low rule-followers through both the direct and the indirect paths. First, low rule-followers decreased their reports in the first-mover position (“setting the stage”), once paired with a high rule-follower rather than a like-minded low rule-follower (Figure 3c, Bayesian multilevel regression, L × mixed, *b* = −0.81, 95% CI = [−1.44, −0.20]). Second, low rule-followers decreased their likelihood to match the number of the first mover (“getting the job done”) when interacting with a high rule-follower (Figure 3d, Bayesian multilevel regression, L × mixed, *b* = −1.11, 95% CI = [−2.19, −0.04]). In contrast, high rule-followers did not significantly change their “setting” and “getting” behavior across team compositions (setting: Bayesian multilevel regression, H: homogeneous vs. mixed, *b* = −0.03, 95% CI = [−0.48, 0.43]; getting: Bayesian multilevel regression, H: homogeneous vs. mixed, *b* = 0.21, 95% CI = [−0.74, 1.15]).

## Discussion and Introduction to Experiment 3

Results of Experiment 2 provide evidence against the social slippery slope hypothesis. Results were consistent with the alternative possibility, namely, that individuals with a stronger propensity to follow rules resist the temptation to engage in collaborative cheating. High rule-followers were both less likely to “set the stage” by misreporting die-rolls, and less likely to “get the job done” by adapting their reporting to match the number reported by their peer. As a result, we observed high levels of collaborative cheating only in dyads consisting of two individuals scoring low on rule following. As soon as one member scored high on rule following, collaborative cheating dropped. Metaphorically speaking, coordinating dishonesty takes two to tango and rule-followers refused to dance.

In Experiment 3, we aimed to test whether this transmission advantage of honesty also generalizes to dyadic interactions in small groups when high rule-followers are in the minority (vs. majority). To this end, we examined collaborative cheating in dyadic interactions within four-person groups containing 0, 1, 3, or 4 individuals scoring high on rule following (and, thus, 4, 3, 1, or 0 individuals scoring low on rule following). Across trials, individuals changed interaction partners, so that in groups with one high rule-follower, low rule-followers were sometimes paired to a high rule-follower and sometimes with a fellow low rule-follower. Based on Experiment 2, we expected the low rule-followers to adapt to the high rule-follower’s (honest) behavior more than the other way around, decreasing group-level collaborative cheating even when only one high rule-follower is present. The other way around, a group of high rule-followers should be less influenced by one low rule-follower in the group.

### Method and Materials

#### Participants and ethics

We invited 195 subjects for a two-part experiment. The study received ethics approval from our Psychology Ethics Board. Subjects provided written informed consent and were debriefed upon completion of the study. The experiment did not involve any deception and subjects were paid for participating at 6.50 €/hr and for their decisions during the experimental tasks. After the first task, participants were scheduled for a second appointment based on the experimental schedule and availability of the subject. Twenty-seven participants failed to show up for their second appointment and did not respond to our contact attempts (dropout rate of 14%). Importantly, first-part behavior of responders and nonresponders did not significantly differ, two-sample *t* test, *t*(193) = −0.01, *p* = .99. Results are based on all participants who took part in both parts of the study (*n* = 168, 128 female, *M*_age_ = 22.45 years, *SD*_age_ = 4.39 years). Because a priori power is difficult to estimate for multilevel repeated-measure designs, sample size was determined based on the number of independent observations per condition (group level) like in Experiment 2.

#### Experimental procedures

Participants first performed the rule-following task (see procedure of Experiment 1) and we split the sample into individuals who scored high on rule following (H) or low on rule following (L) based on the median rule following (median = 20 balls, as in Experiment 2). They were reinvited after 16 days, on average, to perform the dyadic die-rolling task in groups of four. We assigned subjects to groups of four high rule-followers (HHHH groups, *n* = 36), four low rule-followers (LLLL groups, *n* = 36), groups with a majority of high rule-followers (HHHL groups, *n* = 48), or groups with a majority of low rule-followers (LLLH groups, *n* = 48). Prior to participation, participants only knew that the second part of the experiment would take 60 min and that they would interact with other participants.

Each group performed the dyadic die-rolling task for 30 rounds in alternating pairs. In each round, participants were reassigned to a different role (first mover/second mover) and partner based on a predefined schedule that ensured that each group member interacted with each other group member as first mover and second mover once after every six consecutive rounds, and 10 times in total (5 times as first mover, 5 times as second mover), leading to a total of 30 rounds for each subject. Hence, in each round, each participant interacted with one other participant of the group in a team of two. If the team reported to have rolled exactly the same number (a “double”), they got paid according to the worth of the double. This procedure ensured that the payoff function and incentives of collaborative cheating stayed exactly the same across Experiments 2 and 3. As in Experiment 2, Participants did not know about the behavior of their interaction partner in Part 1 (i.e., their private preferences for following or violating rules). Also as in Experiment 2, participants were paid based on their decisions in the rule-following task and one randomly selected round from the dyadic die-rolling task in addition to their participation fee. Participants were paid after the second session. Experimenters could not monitor the behavior of participants in the first or second session and only had information about the sum of money that a participant earned after the second session to be able to pay participants.

#### Analyses

Data were analyzed in the same way as Experiment 2 (see analyses section of Experiment 2), treating the data as hierarchically clustered in subjects (Level 2) and groups (Level 3). Compared with Experiment 2, we observed two independent double reports per round and group and, hence, fitted a second hierarchically clustered random intercept for each group in the Bayesian logistic regressions modeling reported doubles, similar to the regressions modeling die-roll reports (see Equation 1). A frequentist approach based on maximum likelihood estimation (as implemented in the lme4 package in R) did not change the reported conclusions.

### Results

As in Experiments 1 and 2, we found evidence of lying on the sample level. The average die-roll reports were significantly above what would be expected if the sample would have reported honestly, *M* = 3.99, one-sample *t* test, *t*(41) = 7.85, *p* < .001, two sided.

#### Reported numbers

**Figure 4.**
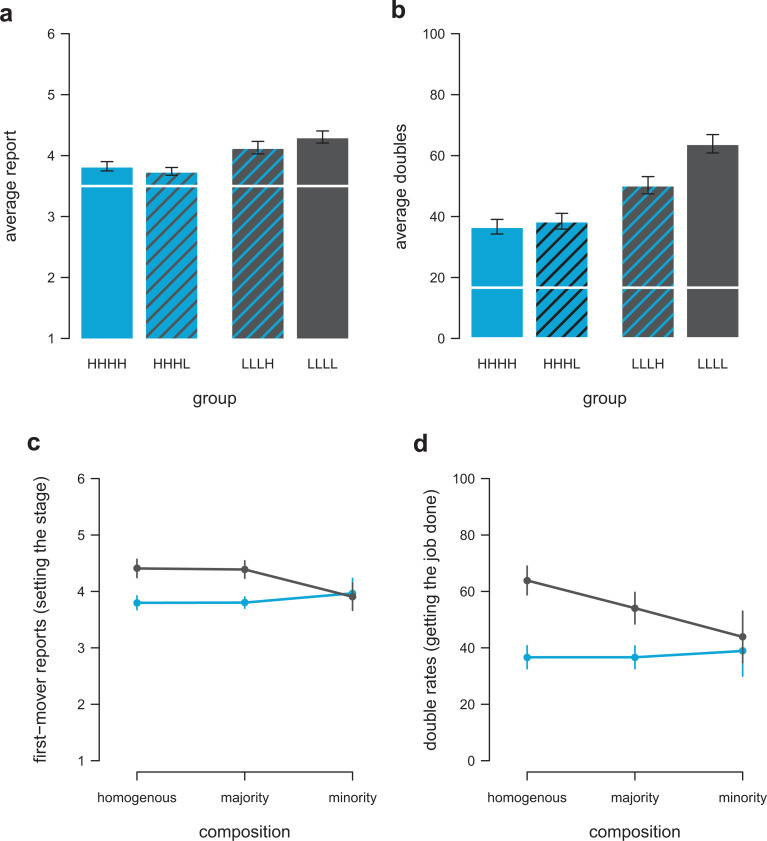
Rule following and collaborative cheating in groups. Average die-roll reports (a) and average percentage of doubles (b) across conditions. HHHH = group of four participants scoring high on rule following, LLLL = group of four participants scoring low on rule following, HHHL = group of high rule-followers with one low rule-follower, LLLH = group of low rule-followers with one high rule-follower. White lines indicate expectation based on chance (i.e., fully honesty). Average die-roll reports of first movers (“setting the stage”; c) and percentage of matching the number reported by the first mover (“getting the job done”; d) of low rule-follower (black) and high rule-followers (blue) when interacting with the same types (homogeneous group), with a majority of same types (majority group), or the opposite types (minority group). Error bars show the standard error of the mean.

Similar to Experiment 2, we found that a group of low rule-followers reported higher numbers than a group of high rule-followers (Figure 4a, Bayesian multilevel regression, HHHH vs. LLLL, *b* = 0.48, 95% CI = [0.15, 0.81]; Table 3). Replacing one high rule-follower by a low rule-follower in a group of high rule-followers did not significantly change die-roll reports (Figure 4a, Bayesian multilevel regression, HHHH vs. HHHL, *b* = −0.08, 95% CI = [−0.39, 0.23]; Table 3). Replacing one low rule-follower by a high rule-follower in a group of low rule-followers also did not significantly change die-roll reports (Figure 4a, Bayesian multilevel regression, LLLL vs. LLLH, *b* = −0.17, 95% CI = [−0.48, 0.14]). We should, however, be cautious with interpreting average die-reports, because the expectation of die-roll reports in mixed groups, even when assuming no social influence between different types, is not simply the average of what we would expect for pure low versus high follower groups (see supplemental material for the derivation). Instead, double rates and individual-level setting and getting behavior allow to derive unbiased conclusions to which degree minorities influence group-level behavior.

**Table 3.** Random-Effects Regression Predicting Die-Roll Reports Based on Group Composition.

			95% CI	
Coefficient	Estimate	*SE*	L	U
Intercept (HHHH groups)	3.82	0.13	3.57	4.07
HHHL groups	−0.08	0.16	−0.39	0.23
LLLH groups	0.30	0.16	0.00	0.62
LLLL groups	0.48	0.17	0.15	0.81
Round	0.00	0.00	−0.00	0.01
σ_level 1_	1.65	0.02	1.62	1.69
σ_level 2_	0.43	0.04	0.35	0.51
σ_level 3_	0.23	0.07	0.07	0.37

*Note.* HHHH = group of four participants scoring high on rule following. LLLL = group of four participants scoring low on rule following. HHHL = group of three high rule-followers with one low rule-follower. LLLH = group of three low rule-followers with one high rule-follower. σ refers to the error term on the individual-decision level (Level 1), subject level (Level 2), or group level (Level 3).

#### Doubles

Whereas a group of low rule-followers reported more doubles than a group of high rule-followers (64% vs. 37%, Figure 4b, Bayesian multilevel regression, HHHH vs. LLLL, *b* = 1.23, 95% CI = [0.53, 1.92]; Table 4), adding one high rule-follower to a group of low rule-followers significantly reduced the likelihood to report doubles (64% vs. 50%, Figure 4b, Bayesian multilevel regression, LLLL vs. LLLH, *b* = −0.64, 95% CI = [−1.28, −0.01]), whereas adding one low rule-follower to a group of high rule-followers did not (37% vs. 38%, Figure 4b, Bayesian multilevel regression, HHHH vs. HHHL, *b* = 0.05, 95% CI = [−0.60, 0.70]; Table 4).

**Table 4.** Random-Effects Logistic Regression Predicting Double Reports Based on Group Composition.

			95% CI	
Coefficient	Estimate	*SE*	L	U
Intercept (HHHH groups)	−0.66	0.26	−1.18	−0.15
HHHL groups	0.05	0.33	−0.60	0.70
LLLH groups	0.59	0.33	−0.06	1.22
LLLL groups	1.23	0.35	0.53	1.92
Round	0.01	0.00	−0.00	0.02
σ_level 2_	0.10	0.07	0.00	0.27
σ_level 3_	0.67	0.10	0.50	0.89

*Note.* HHHH = group of four participants scoring high on rule following. LLLL = group of four participants scoring low on rule following. HHHL = group of three high rule-followers with one low rule-follower. LLLH = group of three low rule-followers with one high rule-follower. σ refers to the error term on the subject level (Level 2) or group level (Level 3). There is no individual-decision level error term because of the logistic regression model.

Experiment 3 also allows us to directly investigate whether the presence of a single participant scoring high on rule following influences how pairs of low rule-followers interact with each other. In groups of low rule-followers, low rule-follower pairs reported to have thrown a double in 64% of the rounds. Pairs of two low rule-followers reported 10% less doubles when one high rule-follower was present in the group. The other way around, a group of high rule-followers reported in 37% of the rounds to have thrown a double. When one low rule-follower was present in the group, the double rate of two high rule-followers, interacting with each other, remained unchanged (37% of the rounds). Thus, the presence of one high rule-follower had a spillover effect on two low rule-followers interacting with each other, resonating with literature showing a social influence advantage for (minority) positions that display societal norms and socially approved practices (Bazarova et al., 2011).

Figure 4c and 4d show that first mover reports (“setting the stage”) and second movers’ matching likelihood (“getting the job done”) were relatively constant for high rule-followers, regardless of whether they were in a group of like-minded high rule-followers, a group with one low rule-follower, or a group of low rule-followers. Accordingly, and in line with the findings from Experiment 2, we did not find statistical evidence that “setting” and “getting” behavior of high rule-followers was influenced by the number of low rule-followers in their group (Figure 4c: setting the stage, Bayesian multilevel regression, *b* = −0.13, 95% CI = [−0.53, 0.26]; Figure 4d, getting the job done, Bayesian multilevel regression, *b* = −0.03, 95% CI = [−0.42, 0.35]). We also found only marginal evidence that first mover die-roll reports of low rule-followers were influenced by the number of high rule-followers in a group (Figure 4c: setting the stage, Bayesian multilevel regression, *b* = 0.36, 95% CI = [−0.03, 0.76]). However, second movers scoring low on rule following systematically decreased their likelihood of matching the first movers report (“getting the job done”) the more participants scoring high on rule following were in the group (Figure 4d, Bayesian multilevel regression, *b* = 0.41, 95% CI = [0.02, 0.78]).

### Discussion

Experiment 3 provided further evidence for the transmission advantage of honesty compared with cheating through a person × situation interaction. Similar to Experiment 2, groups of low rule-followers engaged in more collaborative cheating than groups of high rule-followers. Yet, replacing one low rule-follower with a high rule-follower reduced collaborative cheating in low rule-follower groups, whereas collaborative cheating remained on a similar level when replacing one high rule-follower with a low rule-follower in high rule-follower groups. Already in a minority position, high rule-followers mitigated collaborative cheating and increased honest interactions.

The asymmetric spreading of honesty was mainly driven by low rule-followers lowering their propensity to “getting the job done,” that is, to match the reports of their interaction partner when a high rule-follower was present in the group. The behavior of high rule-followers, however, was largely immune to the composition of the group. Furthermore, the presence of a high rule-follower reduced collaborative cheating among low rule-follower pairs but not the other way around. This finding is noteworthy because high and low rule-followers were not identifiable to one another other than through their behavior, and shows that interactions between high and low rule-followers influenced and spilled over to interactions between two low rule-followers.

## General Discussion

The human capacity for cooperation and coordinated joint action enables groups to perform and create well beyond what people can achieve individually (Gross & De Dreu, 2019). Unfortunately, however, coordinated joint action within groups can take a more destructive form when interacting individuals initiate, endorse, and reinforce deceitful behavior, creating opportunities for collaborative cheating that benefit group members, often at the expense of the broader collective within which groups operate (Goodman, 2015; Gross et al., 2018b). Here, in controlled laboratory settings, we likewise observed substantial levels of collaborative cheating. Across Experiments 2 and 3, dyads reported to have thrown doubles every second round on average; under chance level, a double should occur only every six rounds on average. This finding resonates with previous research on the high prevalence of deceitful behavior in groups (e.g., Cohen et al., 2009; Conrads et al., 2013; Gino et al., 2009, 2013; Kocher et al., 2017; Weisel & Shalvi, 2015).

The puzzle emerging from these and related findings is that collaborative cheating and social slippery slopes exist next to seemingly sticky societal norms for honesty. This puzzle cannot be resolved by assuming that individuals—some more than others perhaps—have social preferences, including concerns for other’s welfare and fairness. Indeed, social preferences would lead people to endorse both societal-level norms of honesty and fairness and reinforce mutually beneficial attempts at collaborative cheating (Fleeson et al., 2014; Heck et al., 2018; Hilbig & Zettler, 2015; Weisel & Shalvi, 2015). Instead, we propose that societal-level norms for honesty can persist and spread because some people are willing to follow rules, even when rule following is personally costly and cheating is in the dyad’s best interest.

Across experiments, we replicated that people differ in rule following. Experiment 1 showed that individuals with higher rule-following propensity are more likely to behave honestly, and Experiments 2 and 3 showed that individuals with higher rule-following propensity are more likely to resist temptations to engage in collaborative cheating with fellow group members. This asymmetric adaption to social influence based on individual differences in rule following creates a transmission advantage of honesty in mixed teams, mitigating the frequency of collaborative cheating. Experiment 3 further showed that honesty not only has a transmission advantage but also is more likely to spread than cheating—pairs of two low rule-followers engaged in less collaborative cheating when their group had a high rule-follower among its members, whereas pairs of high rule-followers did not engage in more collaborative cheating when their group had a low rule-follower among its members. Accordingly, rule following serves as a mechanism for honesty to persist in the face of temptation, reducing the likelihood of collaborative cheating and increasing the probability that honesty within groups is sustained.

Our experiments used a behavioral design with incentivized interactions. This allowed us to explicitly model the cost–benefit structure that underlies societal rules such as honesty. Abiding by rules is often costly, whereas violating rules provides beneficial shortcuts. For example, crossing a red light can save time, avoiding taxes can save money, and cheating on an exam can avoid the effort needed to master the subject. Outside of the laboratory, rules are often both costly and functional—following a rule such as driving on the right (or left) side of the street is in the interest of every individual (Bicchieri, 2005), and waiting in front of a red traffic light reduces the risk of injury. The rule in the rule-following task is rather abstract and we did not provide a reason for following the rule (compared with other studies, such as the classic Milgram experiment in which administering electric shocks was motivated by the researcher’s alleged interest in investigating learning effects). Providing a reason or cover story to motivate rule following decreases experimental control and can create interindividual differences in the degree to which participants believe that following the rule is actually beneficial. The rule-following task, hence, does not disguise its purpose and confronts participants with a clear cost–benefit conflict. Because the rule-following task is quite new, we have little data on its external validity (i.e., to which degree people that score high on rule following also follow societal rules outside of the lab) compared with the more established die-rolling task (for a general discussion on the validity of economic games, see De Dreu & Gross, 2019). Previous research, however, has already shown that rule following predicts norm following in social dilemmas and prosociality (Kimbrough & Vostroknutov, 2016, 2018), is correlated with personal need for structure (Gross & De Dreu, 2017), and can be manipulated through brain stimulation (Gross et al., 2018a). Yet, more focused research is needed to establish a link between rule following in and outside the lab. Accordingly, the strength of our study lies in demonstrating that the rule-following task has high internal validity. It can predict honesty, both on the individual level and in dyadic interactions, and it can explain differences in collaborative cheating.

Similar to the rule-following task, the die-rolling task does not attempt to model all contingencies of dishonesty that exist outside of the lab (e.g., lying detection and subsequent formal or informal punishment). We focused on a situation in which lying remained private knowledge and sanctions were absent. Our behavioral approach has to stay silent on the exact psychological mechanism and possible latent psychological moderators. Rule following as well as cheating could be motivated, for example, by personal image concerns. This would be akin to the idea that rule following is, to different degrees, internalized and that violating rules creates psychological disutility even when rule violations are not observed or sanctioned. Alternatively, rule following and cheating may be motivated by social image concerns, akin to interindividual differences in the sensitivity to psychological demand. Although our experimental procedures tried to increase anonymity and reduce social image concerns, we cannot disentangle whether high rule-followers were motivated by pleasing the experimenter (social image concerns) or because they habitually dislike breaking rules and cheating (personal image concerns). Future studies could also investigate why low rule-followers adapt to high rule-followers in collaborative settings. For example, the behavior of high rule-followers may increase honesty by raising self-awareness or social image concerns in low rule-followers (Grossman & van der Weele, 2016). Future studies could also investigate how personality traits such as honesty–humility, conscientiousness, agreeableness, or ethical convictions (e.g., Wiltermuth et al., 2013) relate to the behavioral rule following.

Both our rule-following and cheating task model a situation in which a rule is at odds with personal self-interest. As such, they measure a similar cost–benefit conflict, which resonates with the results of Experiment 1. How this cost–benefit conflict would be resolved in a social interaction was not obvious and the aim of Experiments 2 and 3. In these experiments, group composition was based on median splits, labeling those scoring below the median as low rule-followers and those scoring above the median as high rule-follower (for a similar approach, see Kimbrough & Vostroknutov, 2016). Splitting individuals based on the median allowed us to create an equal number of low- and high-type teams. The downside is that the exact splitting point can vary across samples, and categorizing individuals based on a continuous measure disregards within-category differences in rule following. It is, therefore, important to interpret results in terms of relative differences in rule following. This notwithstanding, splitting our samples on the median means that half of the sample interacted with the other half. If we assume that people randomly meet, this split makes sense and could explain when honesty norms are followed. Arguably, through a process of self-selection, individuals can also create “dishonest groups” versus “honest groups,” explaining why some corporations and institutions manifest higher levels of rule breaking and corruption than others (Goodman, 2015; Hanna & Wang, 2017). Indeed, forcing people to switch partners prevents collaborative cheating (Abbink, 2004; Gross et al., 2018b).

Our findings combined suggest that already small deviations from median rule following can lead to collaborative cheating and, perhaps, cultures of dishonesty and deception (also see Pryor et al., 2019). Although people frequently cheat and justify rule violations in a self-serving way (Dana et al., 2006; Pittarello et al., 2015; Shalvi et al., 2011, 2015), our findings show that already a minority of rule-followers can foster rule abidance. This nonlinear transmission advantage of honesty may explain how norms that are not governed by formal laws can prevail in society (Gaechter & Schulz, 2016)—displays of norm abidance make honesty more binding and lead to more conformity, outperforming the corrosive effect of norm deviations (see also Bicchieri, 2005).

## Conclusion

The functioning of groups and societies rely on the individual willingness to abide by sets of rules and regulations, to honor trust, and to refrain from cheating (Bicchieri, 2005; Gaechter & Schulz, 2016). Although more in some cultures than others, rule following is often taught as virtuous and promoted by shared narratives within societies (Gelfand et al., 2011). Not all people are rule-followers—we observed substantial variation even in the propensity to follow arbitrary rules. Yet, those who are following rules can serve an important function: They can mitigate the emergence of collaborative cheating. Rule-followers’ selective resilience to social influence may enable norms of honesty and fairness to persist and spread even when cheating is personally beneficial and not formally sanctioned.

## Supplemental Material

Supplemental Material, Gross_Online_Appendix - Rule Following Mitigates Collaborative Cheating and Facilitates the Spreading of Honesty Within Groups Click here for additional data file. Supplemental Material, Gross_Online_Appendix for Rule Following Mitigates Collaborative Cheating and Facilitates the Spreading of Honesty Within Groups by Jörg Gross and Carsten K. W. De Dreu in Personality and Social Psychology Bulletin
