# Supplementary material for: Rule Following Mitigates Collaborative Cheating and Facilitates the Spreading of Honesty Within Groups
Source: Pers Soc Psychol Bull. 2020 Jun 17;47(3):395–409. doi: 10.1177/0146167220927195 (PMC7897793; doi:10.1177/0146167220927195)
Supplement: Supplemental Material, Gross_Online_Appendix - Rule Following Mitigates Collaborative Cheating and Facilitates the Spreading of Honesty Within Groups [file Gross_Online_Appendix.docx]

**SUPPLEMENTARY INFORMATION**

*Rule Following Mitigates Collaborative Cheating* *and*

*Facilitates the Spreading of Honesty within Groups*

1. **SUPPLEMENTARY ANALYSES**

**Expectation benchmarks in the dyadic die-rolling task**

To investigate how actors influence each other in the dyadic die-rolling task, we report die-roll reports, double rates, and individual-level behavior in the first- and second-mover position in the dyadic die-rolling game for Experiment 1 and Experiment 2.

As mentioned in the manuscript, average die-roll reports should be interpreted with caution, since a decrease in average die-roll reports in mixed teams or groups cannot be taken as evidence that social influence across types took place. Instead, changes in double rates provide cleaner evidence for social influence across types. To illustrate this point, we provide theoretical benchmarks here based on the stylized assumption that high rule-followers (H) would always report honestly while low rule-followers (L) always try to maximize expected monetary return.

In low-low pairs (LL) we would expect average die-roll reports of LL = 6. In high-high pairs (HH) we would expect average die-roll reports of HH = 3.5. If we assume that types do not influence each other, we would however not expect average die-roll reports to lie in the middle of these two benchmarks in mixed pairs (HL). For HL-trials (the high-type ‘sets the stage’ and the low-type can ‘get the job done’), we assume that the high-type reports honestly, while the low-type always matches the number reported by the high-type which leads to an expected average die-roll report of (3.5 + 3.5)/2 = 3.5. For LH-trials (the low-type ‘sets the stage’ and the high-type can ‘get the job done’), we would expect the low-type to always report a 6 to maximize potential profits, while the high-type is reporting honesty, which leads to an expected average die-roll report of (6 + 3.5)/2 = 4.75. Taken together, we would expect an average expected die-roll report of HL = (3.5 + 4.75) / 2 = 4.125. This shows that even without assuming that types influence each other, expected die-roll reports of HL pairs would be closer to HH teams than LL teams: HL – HH = 0.625 < 1.875 = LL – HL.

The same holds for groups (Experiment 3). In low-type groups we would expect an average die-roll report of LLLL = 6. In high-type groups we would expect an average die-roll report of HHHH = 3.5. When adding a high-type to a group of low-types, we always have one low-low pair and one low-high pair. For the low-low pair we would expect an average die-roll report of LL = 6 and for the low-high pair we would expect an average die-roll report of HL = 4.125 as shown above, leading to an overall expectation of LLLH = (6 + 4.125) / 2 = 5.0625. When adding a low-type to a group of high-types, we always have one high-high pair and one low-high pair. For the high-high pair we would expect an average die-roll report of HH = 3.5 and for the low-high pair we would expect an average die-roll report of HL = 4.125, leading to an overall expectation of HHHL = (3.5 + 4.125) / 2 = 3.8125. Again, the differences between groups are not equidistant. Even without assuming any social influence, adding a low-type to a group of high-types changes the expected die-roll reports by 3.8125 – 3.5 = 0.3125 points, while adding a high-type to a group of low-types changes the expected die-roll reports by 6 – 5.0625 = 0.9375 points. These calculations illustrate that high-types can affect group level die-roll reports in the dyadic die-rolling task more strongly than low-types without having to assume that one type influences the behavior of the other type.

Yet, this logic does not apply to double rates, i.e. the extent of successful collaborative cheating. In low-low pairs (LL), we would expect a double rate of LL = 100%, whereas in a high-high pair, we would expect a double rate of HH = 16.6%. In mixed teams, we would expect a double rate of 100% when the low-type is the second mover and a double rate of 16.6% when the low-type is in the first position, leading to an average expectation of HL = (16.6 + 100)/2 = 58.3%. Hence, if high and low-types would not influence each other, we would expect a double rate that lies in the middle of the expectation of two high-type and two low-type pairs; HL – HH = 41.7 = LL – HL. In groups, we again would expect a double rate of LLLL = 100% in low-type groups and HHHH = 16.6% in high-type groups. In minority groups we would expect a double rate of LLLH = (100 + (16.6 + 100)/2) / 2 = 79.15% and HHHL = (16.6 + (16.6 + 100)/2) / 2 = 37.45%. Importantly, the expected change in double rates between minority groups and homogenous groups remains constant, if we assume that types do not influence each other in their behavior: LLLL – LLLH = 20.85 = HHHL – HHHH.

**Regression analyses controlling for time distance**

Across all three experiments, participants were invited for two separate experimental sessions. Since we had to take time restrictions of participants into account and sometimes also had to re-schedule appointments due to no-shows or cancelations, we could not keep the time distance between the first and second experimental session constant across participants. In the first experiment, participants came back to the lab for the second part after 8.4 days on average (SD = 2.5, Md = 7, min = 6, max = 14). Experiment 1 reported that rule following was significantly correlated with the reported die-roll outcome. We further ran a regression model that allows us to control for the time (measured in days) between the first and second experimental session that differed across participants. The results reveal that rule following propensity remains a robust predictor of die-roll reports when controlling for the number of days between the first and second lab-visit (b = -0.03, SE = 0.012, t(62) = -2.27, p = 0.027). Time distance was not a significant predictor of die-roll reports (b = -0.01, SE = 0.046, t(62) = -0.28, p = 0.781) and was not significantly correlated with rule following in the first task (Pearson’s r = -0.03, t(63) = -0.239, p = 0.812).

In Experiment 2 and Experiment 3, the second part of the experiment required that participants from the first part would interact with each other. This further required to coordinate on dates across all participants in one session which increased the average time distance between the first and second experimental session. Because of scheduling conflicts, the variance in time distance also increased compared to Experiment 1. In Experiment 2, participants came back to the lab for the second part after 13.5 days on average (SD = 8.2, Md = 14, min = 0, max = 64). In Experiment 3, participants came back to the lab for the second part after 16.2 days on average (SD = 8.1, Md = 15, min = 0, max = 39).

We ran additional regression analyses in which we controlled for the time distance on the individual level. These results are reported in Table S1-S4. Controlling for time distance did not alter the effects and conclusions reported in the main manuscript including the reported planned comparisons (as also shown in Table S1-S4) and the effects of first-mover (setting-behavior) and second-mover (getting-behavior) decisions across types in Experiment 1 (change in ‘setting-behavior’ for high rule-followers when paired with a high vs. low rule-follower: b = - -0.01, 95% CI: [-0.48, 0.47], change in ‘setting-behavior’ for low rule-followers when paired with a high vs. low rule-follower: b = -0.82, 95% CI: [-1.44, -0.19]; change in ‘getting-behavior’ for high rule-followers when paired with a high vs. low rule-follower: b = 0.22, 95% CI: [-0.73, 1.16], change in ‘getting-behavior’ for low rule-followers when paired with a high vs. low rule-follower: b = -1.12, 95% CI: [-2.23, -0.02]) and Experiment 2 (change in ‘setting-behavior’ for high rule-followers when paired with more of their own types: b = -0.03, 95% CI: [-0.21, 0.16], change in ‘setting-behavior’ for low rule-followers when paired with more of their own types: b = 0.22, 95% CI: [-0.03, 0.48]; change in ‘getting-behavior’ for high rule-followers when paired with more of their own types: b = -0.07, 95% CI: [-0.37, 0.24], change in ‘getting-behavior’ for low rule-followers when paired with more of their own types: b = 0.54, 95% CI: [0.13, 0.96]).

**Table S1.** Random-effects regression predicting die-roll reports

based on team composition and controlling for time distance between the first and second part of Experiment 2.

|  |  |  | **95% CI** | |
| --- | --- | --- | --- | --- |
| **coefficient** | **estimate** | **standard error** | **L** | **U** |
| Intercept (HH teams) | 3.44 | 0.20 | 3.04 | 3.83 |
| HL teams | -0.00 | 0.19 | -0.37 | 0.37 |
| LL teams | 0.46 | 0.18 | 0.10 | 0.82 |
| round | 0.04 | 0.01 | 0.01 | 0.06 |
| time distance | -0.00 | 0.01 | -0.02 | 0.01 |
| σ_level 1_ | 1.62 | 0.03 | 1.56 | 1.68 |
| σ_level 2_ | 0.14 | 0.09 | 0.01 | 0.34 |
| σ_level 3_ | 0.45 | 0.07 | 0.31 | 0.60 |
| HL vs. LL teams | -0.47 | 0.19 | -0.84 | -0.10 |

*Note.* HH = pair of two high rule-followers, HL = mixed pair of one person scoring high on rule following paired with one person scoring low on rule following, LL = pair of two low rule-followers. σ refers to the error term on the individual-decision level (level 1), subject level (level 2), or team level (level 3) following equation 1 in the main manuscript. Time distance is measured in days between the first and second experimental session on the individual level.

**Table S2.** Random-effects logistic regression predicting double-reports

based on team composition and controlling for time distance between the first and second part of Experiment 2.

|  |  |  | **95% CI** | |
| --- | --- | --- | --- | --- |
| **coefficient** | **estimate** | **standard error** | **L** | **U** |
| Intercept (HH teams) | -0.93 | 0.53 | -1.97 | 0.10 |
| HL teams | 0.23 | 0.46 | -0.69 | 1.13 |
| LL teams | 1.12 | 0.46 | 0.23 | 2.02 |
| round | 0.04 | 0.03 | -0.01 | 0.09 |
| time distance | -0.00 | 0.03 | -0.05 | 0.05 |
| σ | 1.22 | 0.18 | 0.90 | 1.62 |
| HL vs. LL teams | -0.89 | 0.46 | -1.82 | -0.01 |

*Note.* HH = pair of two high rule-followers, HL = mixed pair of one person scoring high on rule following paired with one person scoring low on rule following, LL = pair of two low rule-followers. σ refers to the error term on the team level. There is no individual-decision and subject-level error term because of the logistic regression model and because reported doubles are measured on the dyadic level. Time distance is measured in days between the first and second experimental session on the individual level.

**Table S3.** Random-effects regression predicting die-roll reports

based on group composition and controlling for time distance between the first and second part of Experiment 3.

|  |  |  | **95% CI** | |
| --- | --- | --- | --- | --- |
| **coefficient** | **estimate** | **standard error** | **L** | **U** |
| Intercept (HHH groups) | 3.99 | 0.16 | 3.68 | 4.29 |
| HHHL groups | -0.09 | 0.16 | -0.40 | 0.23 |
| LLLH groups | 0.28 | 0.16 | -0.02 | 0.59 |
| LLLL groups | 0.53 | 0.17 | 0.19 | 0.87 |
| round | 0.00 | 0.00 | -0.00 | 0.01 |
| time distance | 0.01 | 0.01 | -0.00 | 0.02 |
| σ_level 1_ | 1.65 | 0.02 | 1.62 | 1.69 |
| σ_level 2_ | 0.42 | 0.04 | 0.34 | 0.51 |
| σ_level 3_ | 0.23 | 0.07 | 0.07 | 0.37 |
| LLLL vs. LLLH | -0.24 | 0.16 | -0.56 | 0.07 |

*Note.* HHHH = group of four participants scoring high on rule following, LLLL = group of four participants scoring low on rule following, HHHL = group of three high rule-followers with one low rule-follower, LLLH = group of three low rule-followers with one high rule-follower. σ refers to the error term on the individual-decision level (level 1), subject level (level 2), or group level (level 3).

**Table S4.** Random-effects logistic regression predicting double-reports

based on group composition and controlling for time distance between the first and second part of Experiment 3.

|  |  |  | **95% CI** | |
| --- | --- | --- | --- | --- |
| **coefficient** | **estimate** | **standard error** | **L** | **U** |
| Intercept (HHH groups) | -0.30 | 0.33 | -0.95 | 0.35 |
| HHHL groups | -0.03 | 0.34 | -0.70 | 0.64 |
| LLLH groups | 0.55 | 0.34 | -0.12 | 1.22 |
| LLLL groups | 1.36 | 0.37 | 0.64 | 2.09 |
| round | 0.01 | 0.00 | -0.00 | 0.02 |
| time distance | 0.02 | 0.01 | -0.00 | 0.05 |
| σ_level 2_ | 0.69 | 0.10 | 0.52 | 0.89 |
| σ_level 3_ | 0.58 | 0.14 | 0.29 | 0.86 |
| LLLL vs. LLLH | -0.81 | 0.35 | -1.52 | -0.12 |

*Note.* HHHH = group of four participants scoring high on rule following, LLLL = group of four participants scoring low on rule following, HHHL = group of three high rule-followers with one low rule-follower, LLLH = group of three low rule-followers with one high rule-follower. σ refers to the error term on the subject level (level 2) or group level (level 3). There is no individual-decision level error term because of the logistic regression model.

**Regression analyses using rule-following as a continuous predictor**

We ran further analyses to complement the main results by using the continuous rule-following measure instead of the experimentally pre-defined team and group composition. This allows to further quantify to which degree rule following propensity of the decision maker and the rule following propensity of the partner influences die-reports and double rates. Results are summarized in Table S5-S8. Rule following was a robust predictor of die-roll reports and the likelihood to report a double in the dyadic die-rolling task (similar to the individual die-rolling task in Experiment 1). Importantly, rule following of the interaction partner also predicted the likelihood to report a double across Experiment 2 and 3. For example, with each additional ball that the partner allocated according to the rule in the first part of the experiment, the odds to report a double decreased by 3.1% and 2.6% in Experiment 2 and 3, respectively, in line with the results based on team- and group-level predictors.

**Table S5.** Random-effects regression predicting die-roll reports

based on rule following and controlling for time distance between the first and second part of Experiment 2.

|  |  |  | **95% CI** | |
| --- | --- | --- | --- | --- |
| **coefficient** | **estimate** | **standard error** | **L** | **U** |
| Intercept | 4.020 | 0.206 | 3.615 | 4.424 |
| own rule-following | -0.017 | 0.005 | -0.027 | -0.006 |
| partner’s rule-following | -0.009 | 0.006 | -0.020 | 0.001 |
| round | 0.037 | 0.012 | 0.013 | 0.062 |
| time distance | -0.006 | 0.008 | -0.021 | 0.010 |
| σ_level 1_ | 1.616 | 0.031 | 1.556 | 1.680 |
| σ_level 2_ | 0.141 | 0.093 | 0.007 | 0.344 |
| σ_level 3_ | 0.439 | 0.075 | 0.294 | 0.588 |

*Note.* σ refers to the error term on the individual-decision level (level 1), subject level (level 2), or team level (level 3). Time distance is measured in days between the first and second experimental session on the individual level.**Table S6.** Random-effects logistic regression predicting double-reports

based on rule following and controlling for time distance between the first and second part of Experiment 2.

|  |  |  | **95% CI** | |
| --- | --- | --- | --- | --- |
| **coefficient** | **estimate** | **standard error** | **L** | **U** |
| Intercept | 0.687 | 0.494 | -0.281 | 1.664 |
| own rule-following | -0.032 | 0.012 | -0.057 | -0.008 |
| partner’s rule-following | -0.032 | 0.012 | -0.056 | -0.008 |
| round | 0.043 | 0.018 | 0.007 | 0.079 |
| time distance | -0.004 | 0.014 | -0.032 | 0.024 |
| σ_level 2_ | 0.073 | 0.056 | 0.003 | 0.206 |
| σ_level 3_ | 1.360 | 0.177 | 1.054 | 1.746 |

*Note.* σ refers to the error term on subject level (level 2), or team level (level 3). There is no individual-decision level error term because of the logistic regression model. Time distance is measured in days between the first and second experimental session on the individual level.

**Table S7.** Random-effects regression predicting die-roll reports

based on group composition and controlling for time distance between the first and second part of Experiment 3.

|  |  |  | **95% CI** | |
| --- | --- | --- | --- | --- |
| **coefficient** | **estimate** | **standard error** | **L** | **U** |
| Intercept | 4.615 | 0.150 | 4.327 | 4.911 |
| own rule-following | -0.021 | 0.004 | -0.030 | -0.012 |
| partner’s rule-following | -0.004 | 0.003 | -0.010 | 0.002 |
| round | 0.001 | 0.003 | -0.004 | 0.006 |
| time distance | 0.008 | 0.005 | -0.002 | 0.018 |
| σ_level 1_ | 1.653 | 0.017 | 1.620 | 1.686 |
| σ_level 2_ | 0.394 | 0.042 | 0.318 | 0.482 |
| σ_level 3_ | 0.262 | 0.066 | 0.128 | 0.389 |

*Note.* σ refers to the error term on the individual-decision level (level 1), subject level (level 2), or group level (level 3). Time distance is measured in days between the first and second experimental session on the individual level.

**Table S8.** Random-effects logistic regression predicting double-reports

based on group composition and controlling for time distance between the first and second part of Experiment 3.

|  |  |  | **95% CI** | |
| --- | --- | --- | --- | --- |
| **coefficient** | **estimate** | **standard error** | **L** | **U** |
| Intercept | 0.824 | 0.228 | 0.380 | 1.271 |
| own rule-following | -0.025 | 0.006 | -0.036 | -0.014 |
| partner’s rule-following | -0.026 | 0.004 | -0.034 | -0.017 |
| round | 0.006 | 0.004 | -0.001 | 0.013 |
| time distance | 0.003 | 0.006 | -0.010 | 0.015 |
| σ_level 2_ | 0.357 | 0.053 | 0.256 | 0.461 |
| σ_level 3_ | 0.773 | 0.103 | 0.597 | 1.000 |

*Note.* σ refers to the error term on the subject level (level 2) or group level (level 3). There is no individual-decision level error term because of the logistic regression model. Time distance is measured in days between the first and second experimental session on the individual level.

**2 INSTRUCTIONS**

**Experiment 1/2/3 – Part 1 (Rule Following Task)**

In this part of the experiment, you will decide how to allocate 30 balls between two buckets. Your task is to put each ball, one-by-one, into one of the two buckets: the blue bucket or the yellow bucket.

The balls will appear in the center of your screen, and you can allocate each ball by clicking and dragging it to the bucket of your choice.

For each ball you put in the blue bucket, you will receive 5 cents, and for each ball you put in the yellow bucket, you will receive 10 cents.

The rule is to put the balls in the blue bucket.

Your payment for this part of the experiment will be based on the sum of the payments from the blue and yellow buckets.

This is the end of the instructions for this part of the experiment.

Please click 'I understand the instructions' if you understand what has been explained.


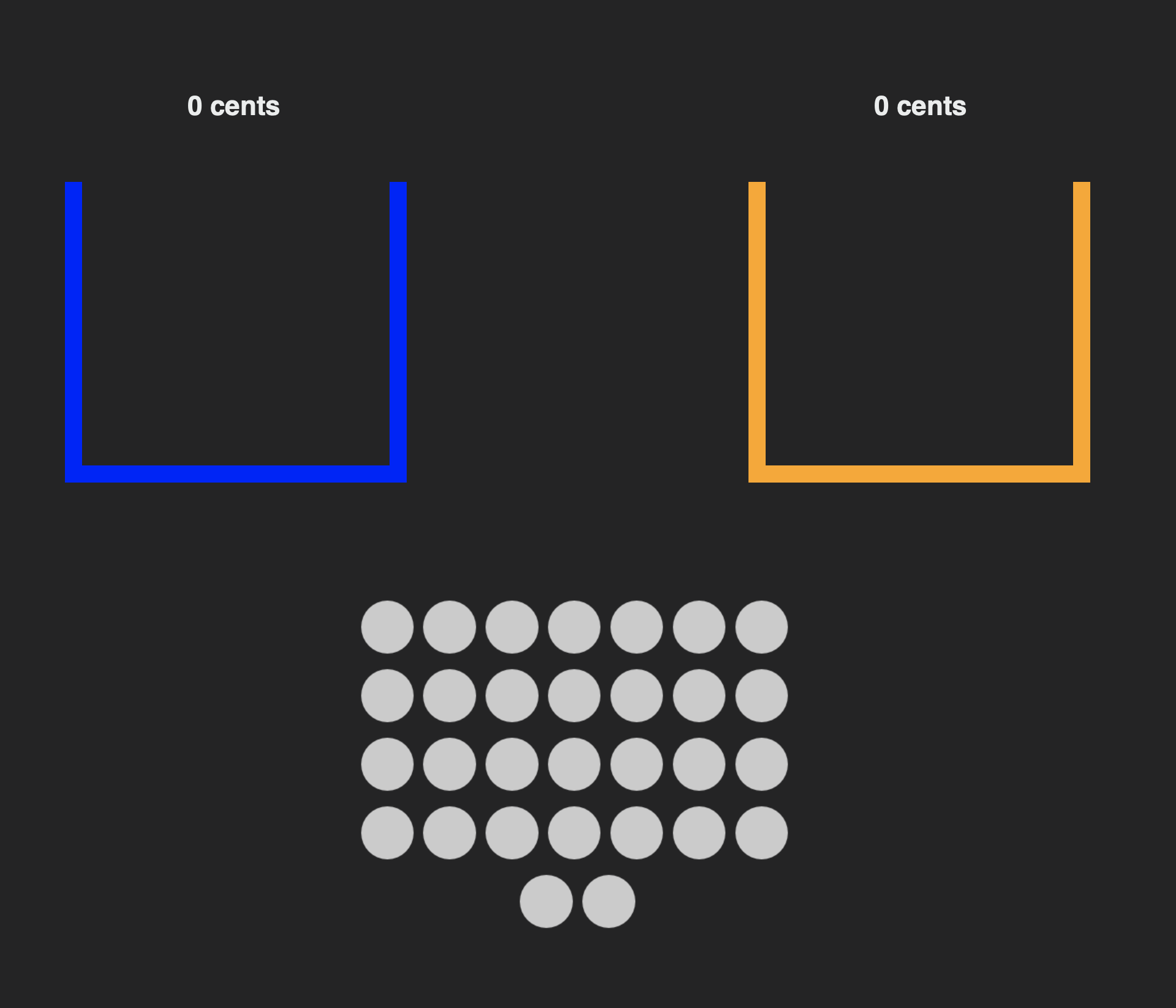


**Figure S1.** Computer Interface of the Rule Following Task

**Experiment 1 – Part 2 (Die Rolling Task)**

This part of the study will consist of 6 rounds.

In each round, you have to roll a die as explained by the experimenter.

Place the die in the cup and cover it with your hands. Then shake the cup, turn it upside down on the table and look at the die-roll outcome.

Then, please report the outcome in the textbox provided on the screen, and click 'submit'.

Once you have clicked 'submit', the next round will begin automatically.

Your payoff will be determined by the result that you report.

Specifically:

if you report a 1 you will earn 0.50 euros,

if you report a 2 you will earn 1 euro,

if you report a 3 you will earn 1.50 euros,

if you report a 4 you will earn 2 euros,

if you report a 5 you will earn 2.50 euros,

if you report a 6 you will earn 3 euros.

At the end of the study, one round will be randomly selected and you will be paid according to the outcome you reported in that round.


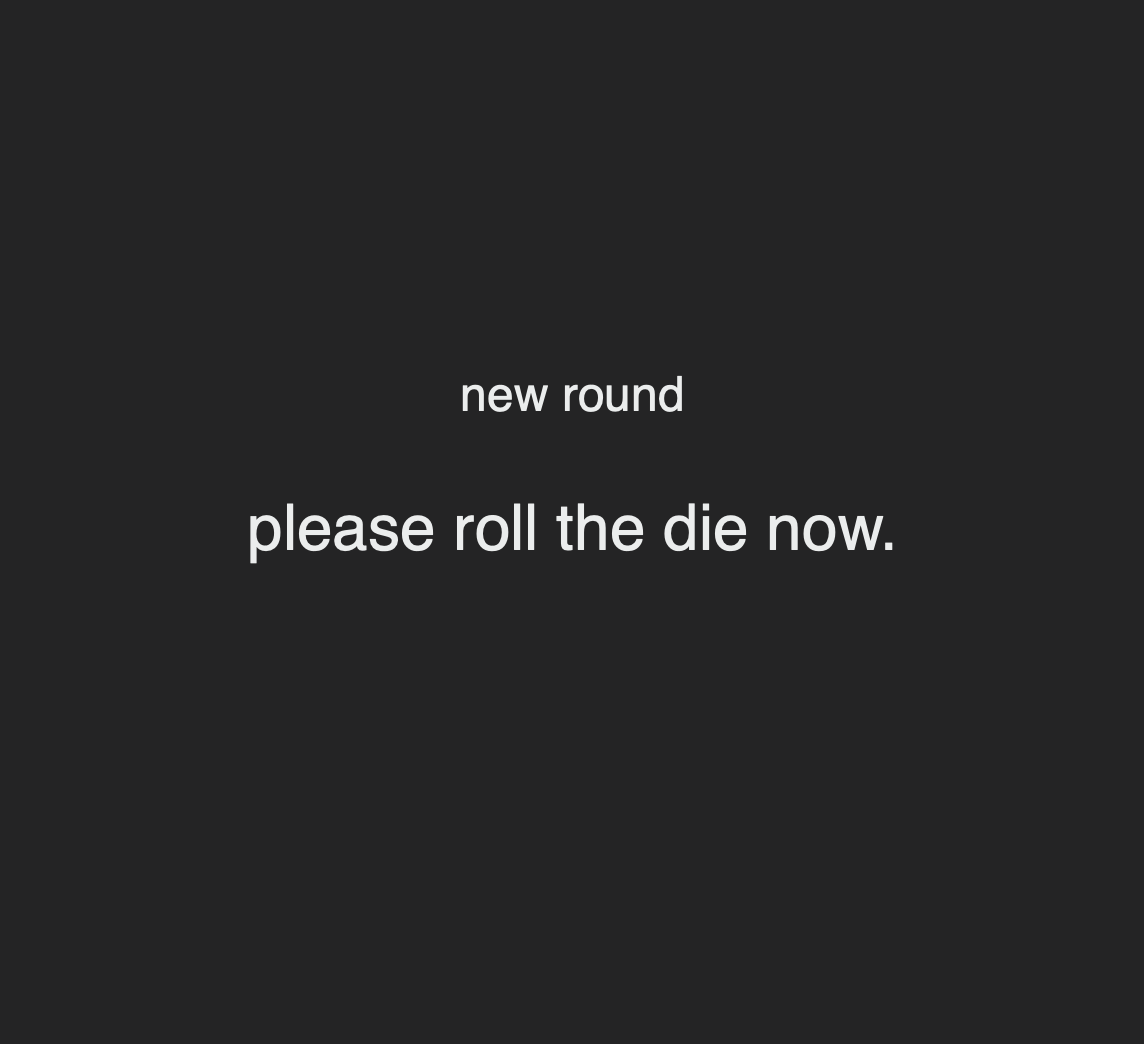

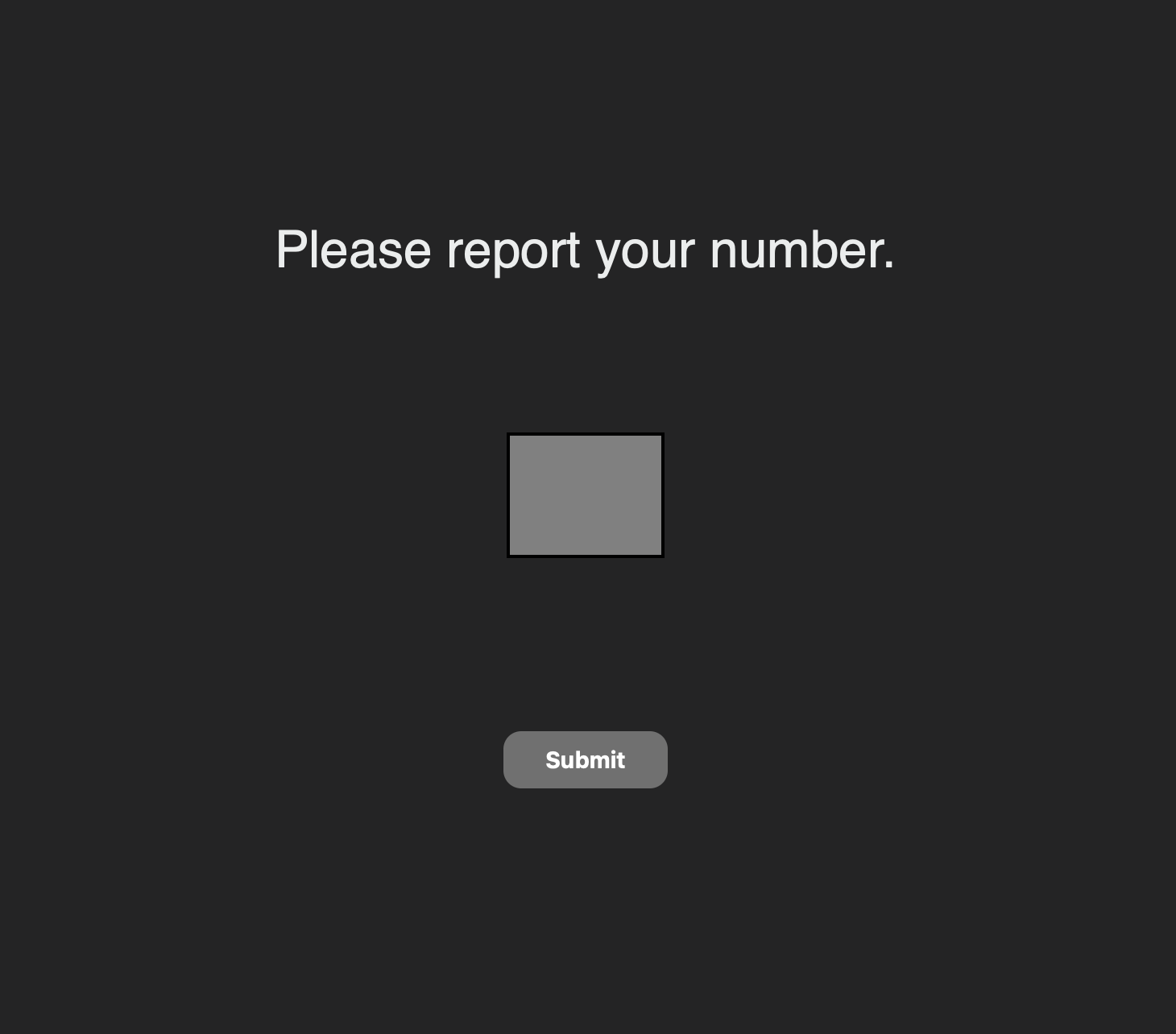


**Figure S2.** Computer Interface of the Die Rolling Task

**Experiment 2 – Part 2 (Dyadic Die Rolling Task)**

*– page 1 –*

This part of the experiment will be performed in pairs.

You are therefore paired with another participant and you will be completing this part together, at the same time.

This part consists of 12 rounds. For each round, one of you will be the 'first mover' and the other will be the 'second mover'.

You will switch roles each round.

For example, if you are the 'first mover' in the first round, you will be 'second mover' in the second round, and so on.

Whether you are the first mover or the second mover will be announced on the screen at the beginning of each round.

As first mover, you will complete the task first and once completed, the second mover will be instructed to complete the task.

*– page 2 –*

The task will be completed as follows:

1. The first mover will be instructed to roll the die, report the outcome, and click 'submit'.

2. Once submitted, the second mover will be shown the number reported by the first mover.

The second mover will then roll the die, report the outcome, and click 'submit'.

3. Once submitted, both participants will be shown both reported rolls for that particular round.

Payment for part II

If both participants report the same number, both will earn additional payment. Payment amounts are as follows:

| first mover report | second mover report | first mover receives | second mover receives |
| --- | --- | --- | --- |
| 1 | 1 | 0.50 euros | 0.50 euros |
| 2 | 2 | 1 euro | 1 euro |
| 3 | 3 | 1.50 euros | 1.50 euros |
| 4 | 4 | 2 euros | 2 euros |
| 5 | 5 | 2.50 euros | 2.50 euros |
| 6 | 6 | 3 euros | 3 euros |

If the outcomes reported do not match, you will not earn additional payment for that round.

*– page 3 –*

At the end of the study, one of the 12 rounds will be chosen at random, and you will be paid according to the outcome in that particular round.

This is the end of the instructions for this part of the study. Please click 'I understand the instructions' if you understand what has been explained.

If you do not understand, please ask one of the experimenters for clarification.

*– page 4 (Comprehension Checks) –*

If the first mover reports a 5 and the second mover reports a 2 what will the first and second mover earn in this round (if selected for payment)?

The first mover will earn 2.50 euros and the second mover will earn 2.50 euros

The first mover will earn 1.00 euros and the second mover will earn 1.00 euro

The first mover will earn 2.50 euros and the second mover will earn 1.00 euro

*The first mover will earn 0.00 euro and the second mover will earn 0.00 euro*

If the first mover reports a 2 and the second mover reports a 5 what will the first mover and second mover earn in this round (if selected for payment)?

The first mover will earn 2.50 euros and the second mover will earn 2.50 euros

The first mover will earn 1.00 euro and the second mover will earn 1.00 euro

The first mover will earn 1.00 euro and the second mover will earn 2.50 euros

*The first mover will earn 0.00 euro and the second mover will earn 0.00 euro*

If the first mover reports a 5 and the second mover reports a 5 what will the first mover and second mover earn in this round (if selected for payment)?

The first mover will earn 5.00 euros and the second mover will earn 5.00 euros

*The first mover will earn 2.50 euros and the second mover will earn 2.50 euros*

The first mover will earn 0.00 euro and the second mover will earn 0.00 euro

**Figure S3.** Computer Interface of the Dyadic Die Rolling Task

**Experiment 3 – Part 2 (Dyadic Die Rolling Task in Groups)**

*– page 1 –*

This part of the experiment will be performed in pairs.

You are therefore paired with another participant and you will be completing this part together, at the same time.

This part consists of 30 rounds. For each round, one of you will be the 'first mover' and the other will be the 'second mover'.

You will switch roles each round.

For example, if you are the 'first mover' in the first round, you will be 'second mover' in the second round, and so on.

Whether you are the first mover or the second mover will be announced on the screen at the beginning of each round.

As first mover, you will complete the task first and once completed, the second mover will be instructed to complete the task.

*– page 2 –*

The task will be completed as follows:

1. The first mover will be instructed to roll the die, report the outcome, and click 'submit'.

2. Once submitted, the second mover will be shown the number reported by the first mover.

The second mover will then roll the die, report the outcome, and click 'submit'.

3. Once submitted, both participants will be shown both reported rolls for that particular round.

Payment for part II

If both participants report the same number, both will earn additional payment. Payment amounts are as follows:

| first mover report | second mover report | first mover receives | second mover receives |
| --- | --- | --- | --- |
| 1 | 1 | 0.50 euros | 0.50 euros |
| 2 | 2 | 1 euro | 1 euro |
| 3 | 3 | 1.50 euros | 1.50 euros |
| 4 | 4 | 2 euros | 2 euros |
| 5 | 5 | 2.50 euros | 2.50 euros |
| 6 | 6 | 3 euros | 3 euros |

If the outcomes reported do not match, you will not earn additional payment for that round.

*– page 3 –*

Importantly four participants will do this task at the same time.

Across rounds, you may not only switch first-mover and second-mover roles, but you are also randomly paired with one of the other three participants.

Hence, in round 1 you may interact with a different person than in round 2, and so on.

In each round, you can only interact with one of three other participants.

You will interact with each other participant multiple times, but you will not know with whom you interact exactly in each round.

*– page 4 –*

At the end of the study, one of the 30 rounds will be chosen at random, and you will be paid according to the outcome in that particular round.

This is the end of the instructions for this part of the study. Please click 'I understand the instructions' if you understand what has been explained.

If you do not understand, please ask one of the experimenters for clarification.

*– page 5 (Comprehension Checks) –*

If the first mover reports a 5 and the second mover reports a 2 what will the first and second mover earn in this round (if selected for payment)?

The first mover will earn 2.50 euros and the second mover will earn 2.50 euros

The first mover will earn 1.00 euros and the second mover will earn 1.00 euro

The first mover will earn 2.50 euros and the second mover will earn 1.00 euro

*The first mover will earn 0.00 euro and the second mover will earn 0.00 euro*

If the first mover reports a 2 and the second mover reports a 5 what will the first mover and second mover earn in this round (if selected for payment)?

The first mover will earn 2.50 euros and the second mover will earn 2.50 euros

The first mover will earn 1.00 euro and the second mover will earn 1.00 euro

The first mover will earn 1.00 euro and the second mover will earn 2.50 euros

*The first mover will earn 0.00 euro and the second mover will earn 0.00 euro*

If the first mover reports a 5 and the second mover reports a 5 what will the first mover and second mover earn in this round (if selected for payment)?

The first mover will earn 5.00 euros and the second mover will earn 5.00 euros

*The first mover will earn 2.50 euros and the second mover will earn 2.50 euros*

The first mover will earn 0.00 euro and the second mover will earn 0.00 euro
